# 479. Optimizing maternal COVID-19 vaccine antibodies in low birthweight infants: is timing everything?

**DOI:** 10.1093/ofid/ofad500.549

**Published:** 2023-11-27

**Authors:** Alisa B Kachikis, Mindy Pike, Linda Eckert, Alexis L Baranoff, Hye Cho, Amber L Young, Erin Goecker, Alexander L Greninger, Janet A Englund

**Affiliations:** University of Washington Department of Obstetrics & Gynecology, Seattle, Washington; University of Washington, Seattle, Washington; University of Washington, Seattle, Washington; University of Washington, Seattle, Washington; SUNY Upstate Medical University, Syracuse, New York; University of Washington, Seattle, Washington; University of Washington, Seattle, Washington; University of Washington, Seattle, Washington; Seattle Children’s Hospital, Seattle, Washington

## Abstract

**Background:**

COVID-19 vaccines in pregnancy may protect young infants from severe illness via maternally-derived IgG. Transplacental transfer of maternal IgG is thought to be decreased in infants with low-birth weight (LBW). The impact of maternal COVID-19 vaccine in this population is unknown. We aimed to evaluate anti-Spike (S) IgG transfer in LBW versus normal birthweight (NBW) infants.

**Methods:**

In this prospective cohort study among individuals with a singleton pregnancy without detectable anti-nucleocapsid IgG who received at least 2 doses of an mRNA COVID-19 vaccine prior to delivery, we tested paired maternal and cord samples for anti-S IgG. We used linear regression to evaluate associations between LBW (birthweight < 2500 grams), timing of vaccine dose, and anti-S IgG. We included as covariates timing of last vaccine dose, gestational age at delivery, number of doses prior to delivery, and small for gestational age (< 10^th^ percentile) birthweight.

**Results:**

We tested maternal/cord anti-S IgG from 33 LBW and 203 NBW pregnancies. The median gestational age at delivery and birthweight was 34.7 weeks/1925 grams for LBW infants compared to 39.4 weeks/3375 grams for NBW infants. Median maternal anti-S IgG was 6128 BAU/mL (IQR:2223,11722) and 1260 BAU/mL (IQR:519,7307) for LBW and NBW infants, respectively. Median cord anti-S IgG was 4585 BAU/mL (IQR:2124,12721) and 1734 BAU/mL (IQR:876, 8812) for LBW and NBW infants, respectively (Figure 1). After adjustment for covariates including vaccine dose timing, there was no difference between cord anti-S IgG concentrations of LBW and NBW infants (beta: -0.01; 95% confidence interval [CI]: -0.68,0.65; p=0.97). As time between last dose and delivery increased, cord anti-S IgG concentrations significantly decreased (beta: -0.0.03; 95% CI: -0.05,-0.02; p < 0.01; Figure 2b). In contrast, cord:maternal IgG ratios significantly increased with greater time between last vaccine dose and delivery (beta: 0.01; 95% CI: 0.01,0.02; p< 0.01; Figure 3).

**Figure:**
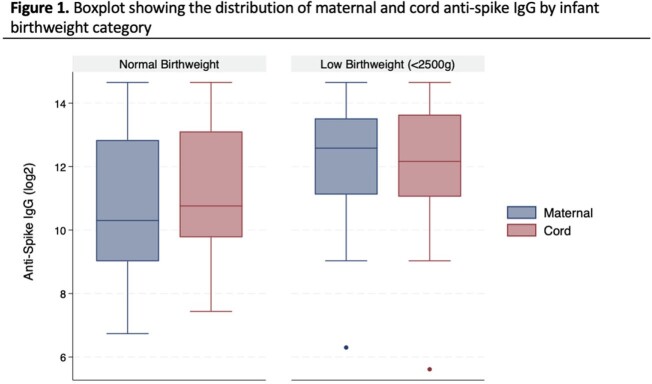

**Figure:**
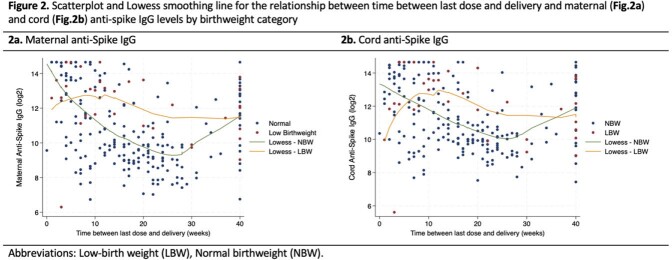

Figure 3

**Figure:**
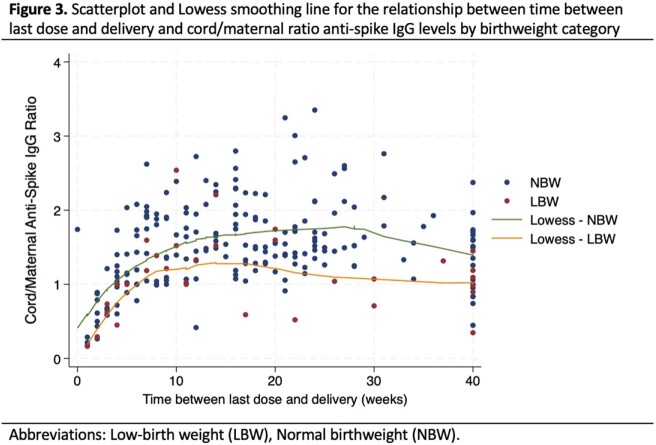

**Conclusion:**

Maternal IgG levels may be more important predictors than infant birthweight for SARS-CoV-2 cord IgG concentrations. Policy regarding timing of COVID-19 vaccine during pregnancy should consider pregnancies at risk for LBW infants.

**Disclosures:**

**Alisa B. Kachikis, MD, MSc**, Merck: Grant/Research Support|Pfizer: Grant/Research Support **Mindy Pike, PhD**, Merck: Grant/Research Support **Alexander L. Greninger, MD, PhD**, Cepheid: central contracts|Hologic: central contracts|Janssen: central contracts|Novavax: central contracts|Pfizer: central contracts **Janet A. Englund, MD**, Ark Biopharma: Advisor/Consultant|AstraZeneca: Advisor/Consultant|AstraZeneca: Grant/Research Support|GlaxoSmithKline: Grant/Research Support|Meissa Vaccines: Advisor/Consultant|Merck: Grant/Research Support|Moderna: Advisor/Consultant|Moderna: Grant/Research Support|Pfizer: Advisor/Consultant|Pfizer: Grant/Research Support|Sanofi Pasteur: Advisor/Consultant

